# Care Poverty Within the Home Space: Exploring the Emotional Experiences of Unmet Care Needs

**DOI:** 10.3389/fsoc.2021.637799

**Published:** 2021-04-29

**Authors:** Tiina Sihto, Lina Van Aerschot

**Affiliations:** Department of Social Sciences and Philosophy, University of Jyväskylä, Jyväskylä, Finland

**Keywords:** care, care poverty, emotions, Finland, home, home care

## Abstract

Older adults face inequalities in care. The concept of care poverty has been developed to point out how unmet care needs are not just an individual issue but a phenomenon linked to social and economic disadvantage and societal inequality. In this paper, we approach the question of care poverty by focusing on its intertwinement with emotions and the home space. We analyze how the presence, or more commonly absence, of care shapes interviewees’ descriptions of emotional experiences tied to the home space. Our data consists of 12 semi-structured interviews conducted in 2019 and 2020 with customers of outreach work for older adults in Finland. These customers typically face a situation that can be characterized as care poverty: their care needs are not (or have not been) met by either the service system or informal sources. When analyzing the data, we followed the guidelines for thematic analysis proposed by Braun and Clarke. Our analysis shows how care and lack of care transform interviewees’ emotional connections with the home space, highlighting particularly three main themes: insecurity, isolation and belongingness. Our analysis reveals how lack of care can transform the home into an unsafe place; a space characterized by isolation, or a space where one sometimes ceases to feel at ease or “like oneself.” The emotional experience of home and being adequately cared for is also tied to the sense of (not) belonging to a place. Based on our analysis, we argue that as an experience, care poverty is not just about individual unmet needs and/or a scarcity of resources at the societal level; it should also be understood as deeply relational—born and shaped in interactions (or the lack of interactions) among people, and lived in and through relationships with others. Furthermore, we highlight the importance of a sense of belonging to the feeling of being adequately cared for.

## Introduction

Older adults face inequalities in care. Having one’s care needs met depends on the availability of informal care from one’s family and friends, the accessibility and affordability of care services from the public or private sectors, and/or one’s own resources to access care services. A person has unmet care needs when help, support or care are needed but the care received, whether formal or informal, is inadequate (Vlachantoni, 2019; Kröger 2010; Kröger et al., 2019).

While it is relatively easy to agree on a definition of the most basic needs—i.e. food, clothing, accommodation, and hygiene—the proper and suitable way to respond to those needs is open to debate. When the scope of needs extends beyond those basic necessities, views about what should count as care needs differ between individuals and societies. It is contested, for example, to what extent frail persons’ social and emotional needs should be recognized as care needs and taken into consideration in publicly subsidized care provision. What counts as care needs, and what constitutes an adequate level, means, and way of meeting those needs, varies historically, culturally, and politically (Godfrey and Callaghan, 2000).

The concept of care poverty has been developed to point out that unmet needs are not just an individual issue but a phenomenon linked to social and economic disadvantage and societal inequality. The concept was coined by Teppo Kröger (2010) to refer to single mothers’ lack of childcare resources. Later the concept has been used in research on care of older people (Kröger et al., 2019). Recently Hill (2021) has conceptualized care poverty as absolute (complete unmet need) and relative (some unmet need, inadequately met needs or poor quality care), stressing the importance of clarifying the threshold(s) that define care poverty.

Previous empirical research has shown that having a low income, living alone, and having poor subjective health status predicts unmet care needs (Kröger et al., 2019). Furthermore, unmet needs are often related to loneliness, social isolation, and an inability to take part in social activities or move around outside of one’s home (Blake et al., 2017). However, care poverty is not necessarily due to a lack of financial resources or social networks; other factors, such as difficulties in obtaining information about available services, can also cause care poverty [(removed to protect author/s anonymity)]. As noted by Hill (2021), the concept of care poverty opens the space to discuss what (good) care means, and what level and type of unmet care needs may be considered as intolerable in the society.

In this paper, we approach the question of care poverty by focusing on its intertwinement with emotions and the home space. We analyze 12 semi-structured interviews with older people who are customers of outreach work for older adults in Finland. Outreach work targets older adults who have been left without sufficient help or support for their care needs. The objective of outreach work is to help these people to access services and find additional help, for example from third-sector organizations. Outreach work departs from the person’s individual needs and aims to empower that person as well as ensuring adequate care. The customers of outreach work for older adults typically face a situation that can be characterized as care poverty: they have (had) care needs that have not been met by either the service system or informal sources (see Pietilä and Saarenheimo, 2018).

As all our interviewees were currently living in their own homes, in regular housing (rather than e.g. service housing or residential care), home was a central place in their accounts of (not) receiving adequate care. In this article we analyze how the presence, or more commonly absence, of care shaped our interviewees’ descriptions of emotional experiences tied to the home space. In addition, we aim to further develop the concept of care poverty theoretically, as it has previously mainly been used in quantitative research [e.g Kröger et al., 2019; however see Kröger 2010; Hill 2021].

## Emotions, Home, and Care

Social and emotional needs are often overlooked in care research, which has mostly focused on exploring the fulfillment of caring tasks (caring for) while often ignoring the emotional and relational aspects of care (caring about) (Larkin et al., 2019). Yet the human world is constructed and lived through emotions. Our understanding of the world’s workings will be incomplete if we ignore a key set of relations through which lives are lived and societies made (Anderson and Smith, 2001).

Emotions are a central part of the everyday of care and how caring relationships are lived and organized. Caring and being cared for can encompass a variety of emotions, such as love, empathy, gratitude, compassion, hate, resentment, bitterness, and regret [e.g. Bondi, 2008; Milligan, 2005; Sihto, 2018]. For decades, research on the sociology of emotions has challenged the idea that emotions are exclusively internalized, subjective mental states; it sees them as also shaped by the broader sociocultural context. The socio-spatial mediation and articulation of emotions adds another layer to this: emotions are shaped by not only the social but also the spatial context in which they take place (e.g. Bondi et al., 2007). In this article, our understanding of emotions follows Bondi et al. (2007, 3), who define emotions as “relational flows, fluxes and currents, in-between people and places rather than things or objects to be studied or measured.”

In caring relationships, the emotions felt by both caregiver and care recipient are also intertwined with how they experience the intimate and institutional spaces where the practices of care take place (e.g. Milligan, 2001; Sihto, 2018; Herron and Skinner, 2013). Space and place shape our emotions, and vice versa—emotions are always tied to the place(s) and space(s) where they occur (Davidson and Milligan, 2004). Spaces and places are not only material but also symbolic, and they carry different meanings at different times. Our experiences and feelings of/in place are constantly (re)shaped by interactions between and among people and environments, by the unfolding of life course(s), and by the destabilizing events that take place during the life course, such as births, bereavements, and the beginnings and endings of relationships (Bondi et al., 2007, 1). Thus spaces and places are relational, and due to this relationality they are in constant flux.

Home is a complex site of study, as the home environment has multiple meanings. Home is not only a dwelling; it is also a place of social interaction, ease and relaxation, personal identity, and privacy (Mattes et al., 2019). At home, past experiences and memories, as well as anticipations of the future, are intertwined with current experiences and emotions attached to the concrete and symbolic aspects of home. Home as a place carries traces and embodies memories of the different people who have lived or passed through it, and thus functions as a reminder of past identities and relationships (Twigg, 2000; Urry, 2005). The presence of familiar surroundings, possessions, and objects helps to give home a personal meaning, and can reinforce one’s sense of self.

Due to the familiarity and privacy of home, homes are often seen as “safe havens” from the threats of the outside world. Feeling safe, secure, and in control of one’s surroundings can also facilitate one’s ability to successfully age in place (Milligan, 2009). However, the privacy for which home is valued can also lead to abuse, neglect, personal unhappiness, loneliness, and social exclusion. The lives of people with (physical) care needs are not necessarily voluntarily home-centered; due to accessibility issues, such people may become involuntarily home-bound, a state characterized by isolation and boredom (Twigg, 2000). This home-boundedness and spatial exclusion can in turn have adverse consequences for older people’s social participation (e.g., Schwanen et al., 2012).

Sixsmith (1986) divides the meaning of home into three dimensions: the physical home (e.g., the architecture, physical structure, furniture, and location of home), the social home (the presence of and relationships with other people in the home space), and the personal home (the relationship between places and the attributes and processes of the self). Cloutier et al. (2015) add the dimension of the emotional home, i.e. how people connect emotionally to the home space, and how home becomes an extension of “one’s own desires, feelings, hopes, and actions” (Oswald and Wahl, 2005, 29 see also; Milligan, 2005). In this article, we focus on the emotional home, although this is inevitably also connected to the other three dimensions of home.

## Data and Methods

In this paper, we analyze 12 semi-structured interviews conducted in 2019 and 2020 with customers of outreach work for older adults in three Finnish cities. Outreach work departs from the customer’s individual needs and aims to create a trusting relationship, ensure adequate care, and empower the customer. The workers help their customers to access services or find additional help, for example from third-sector or voluntary organizations. The customers of outreach work are often older people with a history of social, economic, and health-related disadvantage and/or people who have encountered a disruptive life event, such as the death of a spouse or a rapid health decline.

Our interviewees’ ages ranged from under 60 to over 90 years. Two were men (Matti and Elias), and 10 were women. At the time of the interviews, all lived alone, except one (Anja) who was a spousal carer. All were currently living in regular housing. Their backgrounds were extremely diverse. Some had had heteronormative life courses that had become disrupted relatively recently (e.g. by widowhood), while others had lived alone throughout their lives. For some, disadvantage in old age was the result of lifelong marginality due to (mental) health problems, poor education, unemployment/short-term employment, low income, and/or social isolation. Others had begun to experience societal disadvantage only relatively recently. The interviewees’ characteristics are presented in Table 1.

**TABLE 1:** Interviewees.

Pseudonym	Age group	Family situation	Help and services received (in addition to outreach work)	Services purchased
Matti	Under 60	Single, no children	None	None
Taina	60–69	Divorced, no children	Home help with medical issues and cleaning	None
Anita	60–69	Single, no children	None	None
Anja	60–69	Married (spousal carer), children	Care services for spouse, cleaning services	Cleaning and renovation services
Maria	70–79	No mention of (past) spouse(s), only child deceased, grandchildren	Home help with showering and medical issues; voluntary help with grocery shopping and pharmacy	None
Elias	70–79	Single, no children	None	None
Saara	80–89	Widowed, children and grandchildren	Home help for showering and medical issues	Cleaning services
Sylvi	80–89	Single, no children	None	Cleaning services
Maija	80–89	Divorced, children and grandchildren	Home help with medical issues	None
Raili	80–89	Widowed, children and grandchildren	None	Cleaning services
Elina	80–89	Widowed, children	None	None
Anne	90 or over	Divorced, children	Home help with medical issues	None

The interviews were conducted by the authors in three of Finland’s largest cities: Helsinki, Espoo, and Tampere. Due to these cities’ geographical contexts, they display particularities compared with, for example, aging in rural contexts (cf. Herron and Skinner, 2013). The interviews in 2020 were done before the outbreak of COVID-19 pandemic in Finland, so all the interviews were done face-to-face. The interviews were conducted in interviewees’ homes, in spaces provided by outreach organizations, or in public places such as cafés. This initially also shaped the interview dynamics. The interviews conducted in interviewees’ homes could be characterized as more intimate and affective; interviewees could refer during the interview to specific places and material artifacts (such as photographs) in their homes.

The interviews were semi-structured, and the duration ranged from approximately one to two hours. At the beginning of the interviews we asked questions about the interviewees’ life course, followed by questions about their need and use of care services. When analyzing the data, we followed the guidelines for thematic analysis proposed by Braun and Clarke (2006). The preliminary coding of the data was done by the first author noting the expression of different emotions in relation to different places and spaces. At this stage, the broad focus was on how interviewees described their emotional experiences concerning (lack of) care in various intimate and institutional spaces and places.

For several of our interviewees the home was the most central place for (not) receiving care. After discussing the more specific theoretical framework of the article, the authors therefore decided next to focus on the home space in order to explore it in more depth. This was followed by thematization, which focused on the different emotional experiences tied to the home space. The first author has been responsible for writing the preliminary analysis. After this, the second author supplemented the analysis, particularly regarding the interviews that were conducted by her. The interviews contained affective intensities that could be seen as “glowing” in the data (see Lahti, 2018; see also; MacLure 2013). These data "hotspots" could not always be read from the written interview transcripts. Therefore, both the authors' output was essential in making sense of and analyzing the interview encounters as more than just written interview transcripts.

“Reading” emotions in interview situations and from interview transcripts raises the question of how well the researcher can truly make sense of the interviewee’s emotional states, and what authority the researcher has to make these interpretations in the first place (see e.g. Laurier and Parr, 2000; Bondi, 2014). We are aware that care is a sensitive topic; in particular, describing one’s experiences of being left without adequate care may be difficult and experienced as stigmatizing. Thus, it is possible that interviewees might self-consciously distance themselves from their most painful experiences (Milligan, 2005). This became particularly evident in interview situations. For example, some interviewees wanted to skip some of the questions related to family histories, past relationships, work careers, or (physical or mental) health. In these situations, we as interviewers gave the interviewees space to talk as much or as little as they wanted.

While conducting the analysis, we were interested not so much in our interviewees’ “true” emotions, but rather in how they described and articulated their emotions during the research encounter. Consequently, we understood the emotions expressed and articulated in interview encounters not only as descriptions of interviewees’ personal experiences, but also as reflections of the ways in which it is culturally acceptable to talk about emotions as part of needing and (not) receiving care: which emotions are considered “acceptable” in which circumstances, and which emotional experiences are kept hidden.

## Results

### Insecurity, Disappointment, and Uncertainty: Being Left Without Adequate Care in the Home Space

As all the interviewees lived in their own homes, home was a central site for receiving, or more often not receiving, adequate care. It soon became apparent that for several of the interviewees, care poverty was not just about the lack of adequate services, but was also related to the feeling of being dismissed (often by services, or by society at large), which sometimes resulted in their “giving up” and coping (or at least trying to) on their own. In the interviewees’ accounts, experiences of lack of care became entangled with experiences of the home and the emotions attached to it. For some interviewees, home had become a place where they no longer felt safe: chores and routines such as cooking, cleaning, and showering had become aspects of daily life that they could no longer manage or were afraid to do by themselves. These interviewees expressed feelings of insecurity and even fear because their deteriorating health and limited physical capabilities had not been met with an adequate level of care.

One interviewee who expressed feelings of insecurity and fear in her home was Saara. She was in her 80s, and at the time of the interview she lived alone. Until recently, Saara’s life course had followed a middle-class, linear trajectory: getting married, raising children, and having a relatively stable work career followed by retirement. Recently, this linearity had been abruptly disrupted by the death of her spouse and the subsequent deterioration of her own physical health. She contrasted her life “before” and “after” by describing how *“[before I] was completely healthy. I took care of [my husband’s] funeral, and the next day I could no longer walk.”* Saara said that immediately after the death of her spouse and the decline in her health, she became eligible for home care services. However, at the time of the interview she had been informed that her application for public home care services had been denied. This had been a source of disappointment, worry, and fear:

I would want [help with] taking a shower, because it is dangerous. I have fallen over many times on this floor, and that’s why I have a safety phone. But they said that if I fall down, then I should just call on the safety phone, because I don’t have to take it off my wrist.

Saara’s unmet care needs were concretely related to showering, which she was afraid to do by herself. She described her fear of falling and having to wait naked on the cold, wet floor, possibly hurt and all alone, for someone to come and help. She described the safety phone as offering very little safety: she had previously fallen in the shower, and had lain on the floor waiting for help to arrive, without knowing when someone would come. Saara also talked about disappointment with the care services she had received earlier. This disappointment was manifested in her relational encounters with home care workers. In her account, the disregard she felt home care workers had for her home manifested a disregard for her well-being:

There is very little discussion [with home care workers], because they don’t have the time. When I got out of the shower and put my clothes on, they just left. So nothing else. Nobody even asks can I take your garbage out [laughs]. But that’s how it is. I hate having to complain.

Saara expressed feelings of disappointment and being dismissed: in her account, home care workers did not have time to talk to her, but came only to take care of one particular task (showering). When that task was done, they left, without showing any interest in or care for her or her physical surroundings. A similar comment was made by Maija, who described how she was still physically capable to do some household chores or walk short distances, but how doing these meant that she was in constant physical pain. Maija felt that her pain was being dismissed, as home care workers would only help her with one task (medical issues) but would not help with the doing of everyday household chores that caused her physical pain, such as taking out the trash. She describes how she has several times asked if *“the girls [home care workers] could take [the trash out] but often they say that they don’t have time.”*

As home can be seen as a representation of the self, these interviewees’ experience of home care as neglecting their homes became entangled with their experience of personal neglect as human beings. This disregard for the home also came to embody the “uncaringness” of home care services as an institution. The negative feelings Saara and Maija expressed about home care services were ultimately linked to their lack of trust in the home care service system, which was often seen as unpredictable, unjust, and lacking transparency (see also Lolich and Timonen, 2020). This also made everyday life in the home space unpredictable, transforming the home into an unsafe space.

Maria too expressed feelings of disappointment with the level of services she received, and felt insecure and isolated at home alone. She was in her 70s and had recently been hospitalized for several weeks because of surgery to repair injuries she had sustained in falls. She had been granted daily home care visits when she first came home from hospital, but at the time of the interview these visits had been reduced to twice a week, on Wednesdays and Fridays. Maria felt that this was not sufficient, as she had to be all alone for several days, from Friday to Wednesday. She described how she could not do household chores because she could not stand up steadily. She used a wheeled walker to move around her apartment, and to go out she needed a wheelchair. She could only go out accompanied, as she did not have the ability to move around using the wheelchair on her own. Maria was constantly afraid of falling at home, and she emphasized that everything she did by herself entailed a risk because she could fall any time.

Maria described feelings of abandonment and loneliness, and also bewilderment and disbelief as she had never expected to end up without sufficient care and help at home. She described her social and psychological needs as no less essential than her needs for practical help due to her physical disabilities:

Home care doesn’t work, speaking as a home care client, it does not meet my needs as it should. They should consider me as a person with both physical and psychological needs. That a human being is a psychophysical person. You must understand that I can’t sit here all alone 24 h a day for several days. For what, five or six days from Friday to Wednesday. It is not good for me. And yes, I might have a visit from a volunteer, but that is with an if. If they have time, someone will come.

Maria described a mismatch between the content and amount of care she received and the content and amount of care she needed, emphasizing the centrality of her social needs. She also described living in constant uncertainty because she had to rely on volunteer help with her shopping and pharmacy visits, but that volunteer help worked in unpredictable ways. She said sometimes the volunteers did not have time to come and she did not know when she would be able to buy food or pick up her prescriptions. In her account, the time she spent at home without home care or volunteer help was constructed as stagnant, “empty time” spent sitting *“here all alone 24 h a day for several days.”* This transformed the home into a place of social isolation and uncertainty, making her life unwillingly home-bound (see Twigg, 2000).

### Home as a Site of Intertwining Spatial, Social and Emotional Isolation

In some cases, the strong policy emphasis on home as the best place to grow old and “aging in place,” and the consequent shift of care from institutional spaces to the home, may also be intertwined with the spatial exclusion of older people, leading to adverse consequences for their social and emotional wellbeing (Schwanen et al., 2012).

In several interviewees’ accounts it became apparent that movement from one place to another was impossible due to issues related to accessibility. Steep hills, too many stairs, and the high price of taxi services prevented interviewees from leaving their homes (cf. Pietilä and Saarenheimo, 2018; see also; Twigg, 2000; Blake et al., 2017). This often transformed home into a site of spatial and social isolation. The interviewees had to find alternative ways to overcome these obstacles in order to find new ways to assisted autonomy.

Sylvi, who was in her 80s, described spatial exclusion and a subsequent decline in social participation because her living surroundings were not accessible to her. She talked about having lived alone for almost her whole life. She described feelings of contentment and happiness when she looked back on the time when she had been able to move around the house and outside the sphere of the home. She recalled an active and independent life of travel, hobbies, and attendance at public lectures, which had been disrupted by the decline in her physical health. Due to her health, she was no longer able to use public transportation or walk ordinary distances. She described having relied on the help of strangers to walk the distance to the library near her apartment, back in the days when she could still do so:

It would be easy to go there [the library], but I haven’t been able to go there anymore either […] I cannot walk the distance […] Sometimes a passerby asked if I needed help. But then if nobody came, then I asked a passerby [for help]. I tried to look for a person who wouldn’t have these [earphones] in their ears. And who don’t have heavy grocery bags to carry. So I can get help for a part of the journey, so that I can lean [on that person] with my arm.

Home had become a place where Sylvi was isolated from the rest of the world. She tried to seek help, when possible, by going out and waiting for a passerby to help her or asking help from people on the street. Sylvi attempted to manage her daily life independently even if she needed help from other people. Out of the interviews, Sylvi's interview was particularly filled with 'glowing' (MacLure 2013) affective intensities and her description of emotions attached to her home were less grim than for some of the interviewees who described home as 'prison-like' space. Even though Sylvi expressed sadness due to social isolation and not being able to move outside the sphere of home, her description of home was also vivid with small joys of everyday life:

I can see the sky, and I can see how the scenery changes, and how leaves come to trees and where they go [...] And I can even hear the wind whisper. I haven't heard that before. [...] There is a bush or a tree, that has become sparse, it has gotten old, somehow the twigs have grown but they have fewer leaves. So I can hear the wind whisper, and I think it's wonderful. I like the sound of wind whispering [laughs].

As the interviewees’ physical condition changed, the meaning of home also changed. For some—such as Maria and Saara, who both lived with a constant fear of falling—home became an unsafe place. Maria was isolated in her home spatially, socially and emotionally, as she could not leave her home without assistance and had to wait for someone to come to her home in order to have company. For some, home was not a site for spatial isolation due to movement from one place to another being impossible due to issues related to physical accessibility of different spaces and places but it had become a mental trap. Anja was a carer for her husband and could not leave her home without having someone to take care of him. Thus Anja was bound to her home, but for different reasons than Maria or Sylvi, who could not leave their homes due to physical disabilities. However, for all these women, home had been transformed into a place of struggle, social and emotional isolation, and entrapment (see also Twigg, 2000). When Anja was asked what she felt were the most burdensome aspects of her life, she replied:

Well, I guess it’s having to take care of the house, having to plan what to eat each day […] I have nightmares even [laughs], once I dreamed that I was there in the bedroom, sleeping by myself, that there was a hole in the roof and water kept pouring in.

Anja described the duties and chores at her house and her inability to share the responsibilities of daily life as the most burdensome aspects of her situation as a spousal carer. Her description of nightmares can be seen as reflecting her fear of the home becoming a space that could no longer be handled or controlled. In another part of the interview Anja described the weight of spousal care leading her to being *“absolutely terrified of this life”* and the temporal confinements that had left her feeling *“like a prisoner”* in her own home. Yet, the feeling of being trapped at home, alone responsible for taking care of everything, was what she described the most difficult to bear with.

### The Complexities of Belonging to a Place

When home becomes a site of care, as was the case for several of our interviewees, the privateness of home meets the publicness of care, which can create tensions in the home space (Milligan, 2001) and transform the sense of belonging to one’s home. These tensions and transformations in belongingness can emerge, for example, when the content or daily schedule of care is not seen as fitting one’s care needs. In this subsection, we explore particularly the interviewees' descriptions of (not) belonging to a place. Following Wood and Waite (2011), we understand belonging as a dynamic emotional attachment and process, which relates to the material and social surroundings of a person.

Raili, who was in her 80s, was one of the interviewees whose sense of home had changed due to care services she received at her home. She saw the home care services she had received as an unwanted intrusion into her daily life (cf. Cloutier et al., 2015). However, it was not the content but rather the timing of the services which she described as invasive, as she described a temporal mismatch between her care needs and the schedules of home care. This temporal mismatch shaped her belongingness to home space. As described by Wood and Waite (2011), belonging is about feeling ‘at home’ and ‘secure’, but it is also about being recognized and understood. For Raili, this lack of understanding and recognition regarding her care needs from the home care services had made her feel less at ease, less “at home,” and less “like herself” in her home:

It was a total circus, somebody was always ringing the doorbell. I got so tired of it […] At home, I wasn’t myself, because I was always thinking that somebody is coming and “now they are coming,” and sometimes [they came] at nine in the evening to give me my evening medicine. I was already in bed and [had] washed myself and everything, and then I had to go to open the door […] I had taken my medicine and everything, and I went [to open the door] in my nightgown.

However, even though all our interviewees were customers of outreach work and had thus faced situations that could be characterized as care poverty, not all of them considered the help and care they had received to be insufficient. Elias, who was in his 70s, had a disability that had affected his life since youth; later in life, he had also faced serious health problems. Despite the misfortunes he recounted, his account was also filled with humor as he laughingly described himself as a *“reprobate.”* He had lived in the same neighborhood for over 50 years and expressed a strong sense of belonging (see May and Muir, 2015). Elias described his daily morning routine, which strengthened his sense of belonging in his neighborhood:

My daily schedule is that I get there [in front of the local shopping mall] around nine in the morning. And we have around 10 old men there, and then we chat there and put the world to rights.

Elias did not identify himself as “care poor,” nor did he describe any lack of services. Rather, he saw the cleaning services he received as sufficient. In his account, central to his experience of having enough care was his sense of belonging—he was familiar with and emotionally attached to the physical and social surroundings in his neighborhood, and if he needed help or care he knew where to find it:

I would say that probably, if I went, if I go to the neighbor and need some help, that I will get it. […] Then I have these connections, I know that if I go to the health center, any time I go, when I have these little health problems. [Last time] I went there and got treatment immediately.

Elias knew where he could get help, and he also trusted the service system and had positive experiences of getting the help and care he needed. Furthermore, he had strong social ties to the neighborhood, and the relational and temporal dimensions of his surroundings seemed to particularly support his sense of belonging (May and Muir, 2015).

However, familiarity and positive identification with one’s home did not necessarily translate into a sense of belonging for all our interviewees. Anita, a woman in her late 60s who had lived with a psychiatric disorder since her youth, had changed her place of residence many times in her life, and she had difficulties creating enduring social ties and contacts. She felt she had had to move due to social pressure and stigma when people in her neighborhood had come to know about her disorder. Anita described being anxious due to continuous uncertainty regarding how other people perceived her due to her disorder.

At the time of the interview Anita had lived in her current apartment for five years, but she felt that there was pressure to start planning to move again. However, she had a good relationship with her doctor, and she felt that her disorder was well managed because she was receiving suitable medication and trusted the doctor. Anita had suffered from difficult insomnia at different times of her life, also recently because she was worried that she would need to move again:

Word is spreading again [about my disorder] and prejudices have come out, and so I am awake at night and think if I have to move again. […] That worries me, and I can’t sleep. […] It’s a kind of fleeing. […] But I have this great doctor here, he’ll keep me sane. It’s just about whether I can stand the pressure and … the shunning, I mean in this neighborhood, if I get to know someone. So, usually I don’t tell [anyone] about my mental health problems.

Anita used the word “fleeing” when talking about the need to get away from the social pressure that she felt emerged when people came to know about her mental disorder. Also, when she said that people were starting to avoid her socially, she used the very strong word karttaa, which means “to shun” and in Finnish indicates being systematically avoided and excluded from the company of others.

However, although she expressed a sense of not belonging to the relational surroundings of her neighborhood, Anita did express a sense of belonging that was tied to her home. Having her own, private home was very important to her. Due to her precarious life history, which included short term work and long periods of hospitalization, home implied something of her own—a “protected place” where she could feel she was away from the scrutiny of her neighborhood, and where she could control her daily routines and decisions about what and whom to allow into the space (cf. Milligan, 2001).

Physically Anita did not need help at the time of the interview, although she needed a wheeled walker to move ordinary distances. She could manage all her daily chores by herself and go shopping at the grocery store close by. She felt that all the services she needed were available and accessible. She said that her problem was social isolation and the experience that she could not trust the friendliness of others, even if she managed to make new acquaintances. As the account of Anita shows, belonging is not a single unitary “thing,” but in order to achieve a sense of belonging, creating a sense of identification not only with one's material but also with relational surroundings is essential (see also May and Muir 2015).

The difficulty of constructing a sense of belonging to one’s social surroundings was also mentioned by Taina. Like Anita, Taina also suffered from a psychiatric disorder. Taina described how she had no *“legitimate life story”* to present, which made it difficult to join social networks and create a sense of belonging:

The stigma [of a mental patient] affects various relations, with family and others. [...] When I, so to say, fell ill back in the days I just had to, if I wanted to get back to society, I had to hide the past [in a psychiatric ward] if I wanted to join others. But I was not good at that. It was easier at some point when I was married and had a family, I could hide in there. [...] I used to call myself a person in the margins, one that moves somewhere on the side. Nowadays it might be that I’m socially excluded.

For both Anita and Taina the experiences regarding social prejudices and being marginalized made it difficult to create a sense of belonging and emotional attachment to their social surrounding or neighborhood. Consequently, for them unmet needs were especially tied to the social; need for company of others, places to go and activities to do.

## Discussion

Our analysis shows the various ways in which care and lack of care transformed our interviewees’ emotional connections with the home space. Often the emotional connection with home space was tied together with the experiences of one’s social home (Sixsmith 1986)—the presence, or more commonly absence, of caring relationships with other people in the home space. Lack of care can transform the home into an unsafe space, a space characterized by isolation, or a space where one sometimes ceases feels at ease or “like oneself.” Feeling safe, secure, and in control of one’s surroundings may concern the home space only, or it may also include the surrounding social and material neighborhood. On the other hand, feelings of abandonment or social exclusion can break or inhibit this sense of belonging. However, even if a sense of belonging is not possible, the boundaries of the home space can constitute a caring space, a “safe haven” from the outside world.

Our focus on the intertwinement of the home space, emotions, and (lack of) care shows how complex and multifaceted the phenomenon of care poverty is. Previous studies on care poverty have mainly focused on the quantitative measurement of activities of daily living (ADL) and instrumental activities of daily living (IADL). However, the ADL-IADL scale does not take into consideration the social and emotional needs of the person with care needs [see (Kröger et al., 2019)]. The care poverty faced by our interviewees was not so much absolute, since all of them received some sort of help and care, but rather relative (Hill 2021), and focused on their social and emotional needs. Based on our analysis, we argue that as an experience, care poverty is not just about individuals’ unmet needs and/or a scarcity of resources at the societal level [cf (Kröger et al., 2019)]. Care poverty should also be understood as deeply relational (on relationality, see Mason, 2004) —born and shaped in interactions (or lack of interactions) among people, and lived in and through relationships with others.

According to Mason (2004, 178), “relational practices may well be warm and supportive [but] they may equally be conflictual, oppressive and exclusionary.” Consequently, not all forms of relationality in caring encounters are experienced as inherently “good.” This became especially apparent when our interviewees described their institutional encounters within the home space, particularly with home care workers. Their experiences of home care workers as “uncaring” reflect the scarce resources and time pressures in home care, the uncaringness of home care as an institution, and the commodification of time in home care [e.g Kröger et al., 2018; Cloutier et al., 2015].

However, we do not argue that experiences of care poverty can be reduced to the relational alone. Rather, the relational is tied to and present in the lack of adequate care. In terms of care services, some of our interviewees received home help with e.g. showering or medical issues, and some purchased services from private service providers (see Table 1). However, for several of our interviewees, the lack or inadequacy of care was felt most acutely in the lack of home care support services, e.g. cleaning or help with grocery shopping. For many, in addition to the feeling of being dismissed, this was one of the main manifestations of care poverty. Some interviewees saw this neglect of the home as a neglect of themselves, as the home can be a representation of the self.

In addition to relationality, we also understand care poverty as tied to experiences of not belonging. For some of the interviewees, the need for and the possible receiving of care had transformed their emotional attachment to their material and social surroundings, changing their sense of belongingness (Wood and Waite 2011). Some of our interviewees described how their experience of home had transformed, making home an unsafe, even a prison-like space or how home no longer felt like home. For some, a sense of not belonging was tied to experiences of being socially excluded, and the main manifestation of care poverty was the lack of social ties and need for the company of others. However, achieving a sense of belongingness and creating identification with one’s relational and material surroundings could also facilitate the feeling of being adequately cared for.

To conclude, policy and service delivery (or the lack thereof) play a part in shaping the emotional experiences of individual actors in society. Emotions that arise from being cared for or lacking adequate care can be seen as tied to the individual’s position within the macro-level system of society: as previous studies have shown, care poverty is often tied to older people’s socioeconomic situation [e.g. (Kröger et al., 2019)]. However, our interviewees’ experiences also reveal the precarity of old age. Retirement, illness, and/or the death of a spouse can mean a sudden change in how one is emplaced in the macro-level system. Research focusing on emotional experiences of care and care poverty within the home space can inform the development of care policies that are contextually sensitive and lead to the potential building of more ethical conditions of care (cf. Herron and Skinner, 2013). Our analysis shows how important relationality and sense of belongingness are for good-quality care in the home space, and how essential the feeling of being cared for is for successful and safe aging in place.

## Data Availability

The anonymized data will be made available 2025. Requests to access the datasets should be directed to tiina.sihto@jyu.fi.

## References

[B1] Anderson K. Smith S. J. (2001). Editorial: Emotional Geographies. Trans. Inst. Br. Geog 26 (1), 7–10. 10.1111/1475-5661.00002

[B2] Blake M. Lambert C. Siganporia Z. (2017). Unmet Need for Care. London: IPSOS Mori. Available at: https://www.ipsos.com/sites/default/files/2017-07/unmet-need-for-care-full-report.pdf (Accessed December 4, 2020).

[B3] Bondi L. Davidson J. Smith M. (2007). in “Introduction: Geography’s ‘emotional Turn’,” in Emotional Geographies. Editors Davidson J. Bondi L. Smith M. (Aldershot: Ashgate), 1–16.

[B4] Bondi L. (2008). On the Relational Dynamics of Caring: a Psychotherapeutic Approach to Emotional and Power Dimensions of Women's Care Work. Gend. Place Cult. 15 (3), 249–265. 10.1080/09663690801996262

[B5] Bondi L. (2014). Understanding Feelings: Engaging with Unconscious Communication and Embodied Knowledge. Emot. Space Soc. 10, 44–54. 10.1016/j.emospa.2013.03.009

[B6] Braun V. Clarke V. (2006). Using Thematic Analysis in Psychology. Qual. Res. Psychol. 3 (2), 77–101. 10.1191/1478088706qp063oa

[B7] Cloutier D. S. Martin-Matthews A. Byrne K. Wolse F. (2015). The Space between: Using 'relational Ethics' and 'relational Space' to Explore Relationship-Building between Care Providers and Care Recipients in the Home Space. Soc. Cult. Geogr. 16 (7), 764–782. 10.1080/14649365.2015.1020336

[B8] Davidson J. Milligan C. (2004). Embodying Emotion Sensing Space: Introducing Emotional Geographies. Soc. Cult. Geogr. 5, 523–532. 10.1080/1464936042000317677

[B9] Godfrey M. Callaghan G. (2000). Exploring Unmet Need: The Challenge of a User-Centred Response. York: Joseph Rowntree Foundation Available at: https://www.jrf.org.uk/report/exploring-unmetneed-challenge-user-centred-response (Accessed February 7, 2020).

[B10] Herron R. V. Skinner M. W. (2013). The Emotional Overlay: Older Person and Carer Perspectives on Negotiating Aging and Care in Rural Ontario. Soc. Sci. Med. 91, 186–193. 10.1016/j.socscimed.2012.08.037 23102752

[B11] Hill T. (2021). Understanding Unmet Aged Care Need and Care Inequalities Among Older Australians. Ageing Soc., 1–30. 10.1017/s0144686x21000222

[B12] Kröger T. (2010). Lone Mothers and the Puzzles of Daily Life: Do Care Regimes Really Matter?. Int. J. Soc. Welfare 19 (4), 390–401. 10.1111/j.1468-2397.2009.00682.x

[B33] Kröger T. Puthenparambil J. M. Aerschot L. V. (2019). Care poverty: unmet care needs in a Nordic welfare state. Int. J. Care Caring. 3 (4).

[B34] Kröger T. Van Aerschot L. Puthenparambil J. M. (2018). Hoivatyö muutoksessa. Suomalainen vanhustyö pohjoismaisessa vertailussa [Care work under change: Finnish care work for older people in Nordic comparison]. Jyväskylä: Jyväskylän Yliopisto, YFI-Julkaisuja. 6.

[B13] Lahti A. (2018). Listening to Old tapes,” In Affective Inequalities in Intimate Relationships, ed. Juvonen T. Kolehmainen M. (London: Routledge), 49–62. 10.4324/9781315107318-4

[B14] Larkin M. Henwood M. Milne A. (2019). Carer‐related Research and Knowledge: Findings from a Scoping Review. Health Soc. Care Community 27 (1), 55–67. 10.1111/hsc.12586 29846020

[B15] Laurier E. Parr H. (2000). Disability, Geography and Ethics. Philos. Geogr. 3 (1), 98–102. 10.1080/13668790008573700

[B16] Lolich L. Timonen V. (2020). Fortunate and Fearful: Emotions Evoked by Home-Care Policies for Older People in Ireland. Emotions Soc. 2 (1), 61–78. 10.1332/263169020x15843025702815

[B17] MacLure M. (2013). Classification or Wonder? Coding as an Analytic Practice in Qualitative Research. Deleuze Res. methodologies 46, 164–183.

[B18] Mason J. (2004). Personal Narratives, Relational Selves: Residential Histories in the Living and Telling. Sociological Rev. 52 (2), 162–179. 10.1111/j.1467-954x.2004.00463.x

[B19] Mattes D. Kasmani O. Acker M. Heyken E. (2019). “Belonging,” in Affective Societies: Key Concepts. Editors Slaby J. von Scheve C. (London: Routledge), 300–309. 10.4324/9781351039260-26

[B20] May V. Muir S. (2015). Everyday Belonging and Ageing: Place and Generational Change. Sociological Res. Online 20 (1), 72–82. 10.5153/sro.3555

[B21] Milligan C. (2005). From Home to 'Home': Situating Emotions within the Caregiving Experience. Environ. Plan. A. 37 (12), 2105–2120. 10.1068/a37419

[B22] Milligan C. (2001). Geographies of Care: Space and the Voluntary Sector. Aldershot: Ashgate.

[B23] Milligan C. (2009). There’s No Place like Home: Place and Care in an Ageing Society. Aldershot: Ashgate.

[B24] Oswald F. Wahl H. W. (2005). in “Dimensions of the Meaning of Home in Later Life,” in Home and Identity in Late Life: International Perspectives. Editors D. Rowles G. Chaudhury H. (New York: Springer), 21–45.

[B25] Pietilä M. Saarenheimo M. (2018). Löydettynä: Etsivä Vanhustyö Ja Ikäihmisten Psykososiaalinen Hyvinvointi. Yhteiskuntapolitiikka 83 (5–6), 573–580.

[B26] Schwanen T. Hardill I. Lucas S. (2012). Spatialities of Ageing: The Co-construction and Co-evolution of Old Age and Space. Geoforum 43 (6), 1291–1295. 10.1016/j.geoforum.2012.07.002

[B32] Sihto T. (2018). Distances and proximities of care: Analysing emotio-spatial distances in informal caring. Emotion, Space Soc. 29, 62–68.

[B27] Sixsmith J. (1986). The Meaning of Home: An Exploratory Study of Environmental Experience. J. Environ. Psychol. 6 (4), 281–298. 10.1016/s0272-4944(86)80002-0

[B28] Twigg J. (2000). Bathing: The Body and Community Care. London & New York: Routledge.

[B29] Urry J. (2005). in “The Place of Emotions within Place,” in Emotional Geographies. Editors Davidson J. Bondi L. Smith M. (Aldershot: Ashgate), 00–00).

[B30] Vlachantoni A. (2019). Unmet Need for Social Care Among Older People. Ageing Soc. 39 (4), 657–684. 10.1017/s0144686x17001118

[B31] Wood N. Waite L. (2011). Editorial: Scales of Belonging. Emot. Space Soc. 4 (4), 201–202. 10.1016/j.emospa.2011.06.005

